# Antibody response to a sterile filtered PPD tuberculin in *M. bovis *infected and *M. bovis *sensitized cattle

**DOI:** 10.1186/1746-6148-6-50

**Published:** 2010-11-09

**Authors:** Bryan Rennie, Lionel G Filion, Nonie Smart

**Affiliations:** 1 Canadian Food Inspection Agency, Mycobacterial Diseases, Ottawa, K2H 8P9, Canada; 2 Department of Biochemistry, Microbiology and Immunology, Faculty of Medicine, University of Ottawa, Ottawa, K1H 8M5, Canada

## Abstract

**Background:**

Bovine tuberculosis, caused by *Mycobacterium bovis*, afflicts approximately 50 million cattle worldwide and is detected by the tuberculin skin test (TST). While it has long been recognized that purified protein derivative (PPD) tuberculin is composed of a mixture of *M. bovis *derived protein components, little is known about the quality, relative quantity and identity of the proteins that make up PPD tuberculin. We manufactured a sterile filtered PPD tuberculin (SF-PPD) from a nine-week-old *M. bovis *culture supernatant in order to characterise the culture filtrate proteins (CFP) which make up *M. bovis *PPD tuberculin and to compare the antibody response of *M. bovis *infected versus *M. bovis *sensitized cattle.

**Results:**

SF-PPD resolved into approximately 200 discrete spots using two-dimensional polyacrylamide gel electrophoresis (2-DE) while fewer than 65 spots could be discerned from 2-DE gels of tuberculin derived from autoclaved culture supernatant. Two dimensional Western blot analyses indicated that sera from *M. bovis *sensitized cattle recognized additional SF-PPD antigens as compared to *M. bovis *infected cattle at seven weeks post infection/sensitization. However, application of a comparative tuberculin skin test resulted in an antibody boosting response to the same set of *M. bovis *CFPs in both the *M. bovis *infected and *M. bovis *sensitized cattle.

**Conclusions:**

We concluded that it is the heat sterilization of the *M. bovis *CFPs that causes severe structural changes to the *M. bovis *proteins. This work suggests that *M. bovis *infected cattle and cattle artificially sensitized to *M. bovis *with an injection of heat killed cells exhibit similar antibody responses to *M. bovis *antigens.

## Background

Bovine tuberculosis, caused by *Mycobacterium bovis *infection, is a major global health threat, with approximately 50 million cattle currently infected worldwide [1]. The primary method used to detect tuberculosis in cattle is the single intradermal test (SIT). Although the SIT is the most widely used diagnostic test for *M. bovis *infections in cattle, little is known about the quality, relative quantity and identity of the proteins that make up purified protein derivative (PPD) tuberculin.

PPD tuberculin is a crude and complex mixture of tuberculo-proteins which has changed little since its conception and original application by Dr. Robert Koch in 1890 [2]. The original tuberculin, Koch's old tuberculin, was prepared from a heat sterilized liquid culture medium containing 8 - 12 week old *M. tuberculosis *(*M. tb*) cultures concentrated to one-tenth the original volume by evaporation [2]. While it has long been recognised that PPD tuberculin is composed of *M. bovis *derived protein components, early efforts to accurately characterize the antigenic components of PPD tuberculin [3] were met with difficulty. In retrospect, interpretation of early findings were likely further complicated by protein denaturing effects of heat and pressure exerted during autoclaving and the absence of effective protein separation and characterization techniques. Fractionation of tuberculin into 3 - 14 antigenic fractions by alcohol fractionation [4,5], column chromatography [6-8], and crossed immunoelectrophoresis [9] resulted in the description of a variety of tuberculo-protein fractions with incompletely defined structural characteristics and/or biological activity such as the Antigen "L" [7] and PPD tuberculin fractions A, B and C [5].

With the advancement of molecular separation techniques and polyacrylamide gel electrophoresis (PAGE) examination of non-heated *M. tb *[10] and *M. bovis *[10,11] culture broths more than 800 tuberculo-proteins are currently described in the literature. Consequently, many of the tuberculin fractions previously described as homogenous entities actually consist of multiple mycobacterial proteins. The current accepted terminology for this complex mixture of tuberculo-proteins is culture filtrate proteins (CFP) and this includes secreted proteins, exported proteins and non-secreted, somatic components which are released into the culture medium due to autolysis, replication and bacterial leakage [12,13]. The protein profile of a CFP set is dependent on many factors including cultivation time, temperature, growth medium and culture agitation [13,14].

Today most laboratories use non-heated *M. bovis *culture filtrates rather than tuberculin for the identification of specific *M. bovis *antigens for use as diagnostic and vaccine candidates [10,12,13,15-17]. Separation and characterization of non-heated *M. bovis *CFPs using molecular techniques such as two dimensional polyacrylamide gel electrophoresis (2-DE), mass spectronomy (MASS-SPEC) analysis and in vitro antigenicity assays has lead to the identification of several, highly antigenic *M. bovis *proteins. However, the antigenic activity of these proteins and their conservation in field-use *M. bovis *PPD tuberculin remains largely unknown.

Analysis of *M. bovis *culture filtrate proteins with two dimensional polyacrylamide gel electrophoresis (2-DE) has indicated that PPD tuberculin is derived from a multitude of tuberculo-proteins [10-13]. Tuberculin manufacturing methods, which include heating and chemical treatment, may alter the structure and antigenicity of the tuberculo-proteins in field issue PPD tuberculin. In accordance with international standards [18] the antigenicity of each new batch of PPD tuberculin, in many laboratories, is routinely measured in animals sensitized with heat killed *M. bovis *cells.

As many of the antigenic *M bovis *CFP have been shown to elicit both cellular and humoral responses in cattle [1,19,20], we hypothesized that immunological differences would be observed between cattle infected with *M. bovis *as compared to cattle artificially sensitized to *M. bovis *with an injection of heat killed cells. Our experiments presented in this manuscript showed that classical, autoclaved PPD was essentially a mixture of peptide fragments which could not be sufficiently resolved by SDS-PAGE, 2-DE or Western blot analysis to characterize the antibody responses of our experimental cattle. *M bovis *sterile culture filtrate from 9-week-old cultures, however, could be satisfactorily resolved by these same methods. We concluded that it was the heat sterilization of the *M. bovis *CFPs that caused the severe structural changes and protein fragmentation to the *M. bovis *proteins. The immunological significance of the protein fragmentation, however, remains to be determined. This work also suggested that *M. bovis *infected cattle and cattle artificially sensitized to *M. bovis *with an injection of heat-killed cells possess similar antibody response to selected *M. bovis *antigens. While this study compares the humoral immune response of *M. bovis *infected and *M. bovis *sensitized cattle, future comparisons of their cellular immune responses may lead an *M. bovis *sensitization method which would provide a consistent immune response similar to that of an experimentally *M. bovis *infected animals. The development of an accurate, non-infectious bovine tuberculosis model would reduce the complexity and bio-containment risks associated with cattle studies involving live *M. bovis*.

## Methods

### Production of *M. bovis *SF-PPD and PPD tuberculin

*M. bovis *AN5 was cultured aerobically at 37 ± 2°C on Reid's synthetic, liquid medium. After nine weeks of incubation, the culture flasks were divided into two groups. Culture supernatant from the first group was inactivated by autoclaving at 121°C and 110 kPa for 45 min. Tuberculin produced from this culture supernatant was referred to as heat killed PPD tuberculin (HK-PPD). Culture supernatant from the second group was separated from the live *M. bovis *cells by filtration through a 3 μm and a 0.8/0.2 μm capsule filters (Pall Corporation, USA) arranged in series. Tuberculin produced from this culture supernatant was referred to as sterile filtered PPD tuberculin (SF-PPD). Both the sterile filtered and autoclaved culture supernatant was confirmed sterile by culture.

Both the sterile filtered and autoclaved culture supernatants were filtered at 0.22 μm and concentrated with 10 kDa tangential flow filtration (TFF) cassettes (Pellicon Cassette, Millipore Corporation, USA). Tuberculo-proteins were precipitated by addition of ammonium sulfate, pelleted by centrifugation, re-suspended in a phenolized phosphate buffer (0.0147% (w/v) sodium phosphate dibasic, 0.076% (w/v) potassium phosphate, 0.5% (v/v) phenol) and de-salted/concentrated with 10 kDa TFF. Further dialysis to remove buffer salts and phenol from both SF-PPD and HK-PPD was accomplished using a 5-kDa TFF capsule filter (Minimate™ TFF System, Pall Corporation, USA).

Protein concentrations were determined using the *DC*™ protein assay following the manufacturer's instructions with bovine serum albumin as standard (Bio-Rad, Mississauga, Ontario) [21].

### PAGE and MASS SPEC analysis of HK-PPD and SF-PPD

One dimensional, vertical, sodium dodecyl sulphate (SDS) PAGE was performed as per Lamelli, with the following specifications [22]. A 6% acrylamide, pH 6.8 stacking gel and a 12% acrylamide, non-linear gradient, separation gel with a pH of 8.8 were used. The acrylamide-bis ratio was 29:1 and gels were cast in either 16 × 20 cm or 8.3 × 7.3 cm sizes with thicknesses of either 0.75, 1.00 or 1.50 mm depending on the application. Electrophoresis chemicals and molecular weight standards were purchased from Bio-Rad Laboratories, Mississauga, Ontario. HK-PPD and SF-PPD samples were placed 1:4 into a reducing sample buffer which contained 2.0% (w/v) SDS, 5.0% (vol/vol) 2-mercaptoethanol, 10% (v/v) glycerol, 0.0625 M tris base (pH 6.8), and 0.005% (w/v) bromophenol blue. Samples were boiled at 100°C for 5 minutes and centrifuged for 5 minutes at 14 000 xg prior to loading. The amount of protein applied to each lane varied with the application and size of the gel. Electrophoresis was performed using either the PROTEAN^® ^II or mini-PROTEAN^® ^III cells (Bio-Rad) at 20 mA constant current per gel. Electrophoresis was stopped when the bromophenol blue tracking dye reached the bottom of the separating gel.

HK-PPD and SF-PPD samples separated by 2-DE were first subjected to iso-electric focusing (IEF) along an acrylamide strip, followed by SDS-PAGE, molecular weight separation in a direction 90 degrees from the IEF. The precast, 17 cm IEF strips had an immobilized pH range of 3-10 (ReadyStrip™; Bio-Rad Laboratories). Isoelectric focusing was carried out using a Multiphor™ II Electrophoresis Unit (Amersham Pharmacia Biotech) and a PowerPac™ HV power supply (Bio-Rad Laboratories, Mississauga, Ontario). IEF strips were re-hydrated and loaded with 300 μL of either HK-PPD or SF-PPD prepared in ReadyPrep™ Squential Extraction Kit Reagent 3 (Bio-Rad Laboratories) with 3 μL of tributyl phosphine and 0.0003% (w/v) bromophenol blue. PPD samples were diluted in de-ionized water according to application and strips were focussed for ~98,000 volt hours.

Prior to the second dimension, IEF strips were equilibrated sequentially for 15 min. at 37°C in first a reduction buffer (0.05M Tris, pH 6.8; 8M urea; 35% glycerol; 2% (w/v) dithiothreitol; 0.3% SDS) followed by an alkylation buffer (0.05 M Tris, pH 6.8; 8 M urea; 35% glycerol; 2.5% (w/v) iodoacetamide; 0.3% SDS). Following alkylation, the IEF strips were loaded into 12% SDS polyacrylamide gels with the pH 3 end of the strip towards the molecular weight marker and overlaid with 0.5% (w/v) low melting agarose containing 0.0001% (w/v) bromophenol blue. Second dimension electrophoresis was performed using the PROTEAN^® ^II cell (Bio-Rad) at 20 mA constant current per gel. Electrophoresis was stopped when the bromophenol blue tracking dye reached the bottom of the separating gel. Electrophoresis chemicals and molecular weight standards were purchased from BIO-RAD Laboratories, Mississauga, Ontario.

Gels were stained with either Comassie Brilliant Blue (CBB) R-250 stain (Bio-Rad Laboratories, Mississauga, Ontario) or with a non-fixing silver stain method described by Shevchenko et al. (1996) [23].

SDS-PAGE and 2DE gels of HK-PPD and SF-PPD were digitized in 8 bit greyscale at 300 dpi with a ScanMaker *i*900 scanner (Microtek). Spots on the 2DE gels were enumerated by PDQuest 7.1.0 2D Analysis Software (Bio-Rad). Following automated enumeration, erroneous and duplicate spots were manually deleted.

Nine spots of interest were excised from a CBB stained 2DE gel of SF-PPD (200 μg; 50 μL of 4 μg/μL). Excised spots were placed in sterile vials and maintained at -80°C prior to submission to the Ottawa Institute for Systems Biology (University of Ottawa) for MASS-SPEC analysis.

### Western blot analysis of *M. bovis *Infected and *M. bovis *sensitized cattle sera

The cattle sera used for Western blots were collected from twenty-four Holstein cross cattle in 2004 during a comparative intradermal tuberculin skin test (CITST) study. CITST methodology was based on recommendations from the World Organization for Animal Health's Manual of Diagnostic Tests and Vaccines for Terrestrial Animals [18]. In this study twelve cattle received a 1.0 mL intra-tracheal inoculation of 1500 CFU of virulent *M. bovis *(field strain 02/1007; CFIA designation) and another twelve cattle received a 1 mL intramuscular injection containing 20 mg of heat killed (autoclaved) *M. bovis *cells (field strain 02/1007, CFIA designation) suspended in 50% mineral oil, 25% lanolin and 25% saline. One negative control animal (cattle # 9246) received a 1 mL injection containing 50% mineral oil, 25% lanolin and 25% saline. *M. bovis *sensitized cattle serum from a previous experiment was used as a positive control (cattle # 893). Cattle were screened with an *in vitro *blood based assay (Bovigam™; Pfizer, Australia) prior to infection/sensitization and based on the manufacturer's cut-off values were determined to be negative for bovine tuberculosis. One-dimensional Western blot analyses were performed on sera derived from three *M. bovis *sensitized cattle (cattle # 003,457, 993) and two *M. bovis *infected cattle (cattle # 103, 107). Two-dimensional Western blot analyses were performed on sera derived from six *M. bovis *sensitized cattle (cattle # 003, 112, 207, 211, 457, 993) and six *M. bovis *infected cattle (cattle # 103, 106, 107, 108, 109, 110).

Blood was collected weekly for a period of twenty weeks from all cattle and the sera was maintained at -80°C. CITSTs were applied to both the sensitized and infected cattle at 7 and 13 weeks post sensitization/infection respectively. The Ottawa Laboratory Fallowfield CFIA Institutional Animal care committee approved all animal use and procedures in these studies. All animals received *ad-libitum *food and water and were handled and cared for in accordance with the regulations prescribed by the Canadian Council on Animal Care. *M. bovis *infection status was confirmed in all cattle inoculated with live *M. bovis *by lesions observed on necropsy, by histological identification of acid-fast bacteria with typical histopathology of mycobacteriosis and by isolation of *M. bovis *on culture. Likewise, all artificially sensitized cattle were confirmed negative for *M. bovis *infection by the same tests.

Western immunoblotting of *M. bovis *sensitized/experimentally infected cattle sera onto either HK-PPD or SF-PPD was performed as per Towbin et al, with the following specifications [24]. Electrophoretically separated HK-PPD and SF-PPD was transferred onto nitrocellulose membranes (0.45 μm pore size, Bio-Rad Laboratories) using a tank style blotting system (Transphor Electrophoresis unit, Hoefer Inc., USA) at 100 volts for 1 hour and blocked for 1 hour at 37°C in 7.4 pH, Tris buffered saline (TBST) (0.02 M Tris, 0.8% (w/v) sodium chloride, 0.02% (w/v) potassium chloride, 0.3 (v/v) Tween 20). The nitrocellulose was incubated overnight at room temperature in either *M. bovis *sensitized or infected bovine sera diluted 1:200 in TBST. Nitrocellulose was washed with TBST, incubated for 2 hours at room temperature with alkaline phosphatase conjugated rabbit anti-bovine IgG (Sigma-Aldrich Canada Ltd., Oakville, Ontario) diluted 1:5000 in TBST. Phosphatase substrate (5-bromo-4-chloro-3-indolyl-phosphate/nitroblue tetrazolium, (Mandel Scientific Company Inc., Guelph, Ontario) was applied to the nitrocellulose for 10-15 minutes at which time the reaction was stopped with the addition of de-ionized water. Western blots were examined visually and were also digitized in 8-bit greyscale at 300 dpi with a ScanMaker *i*900 scanner (Microtek).

## Results

### HK-PPD and SF-PPD examination by SDS-PAGE

**Figure 1:**
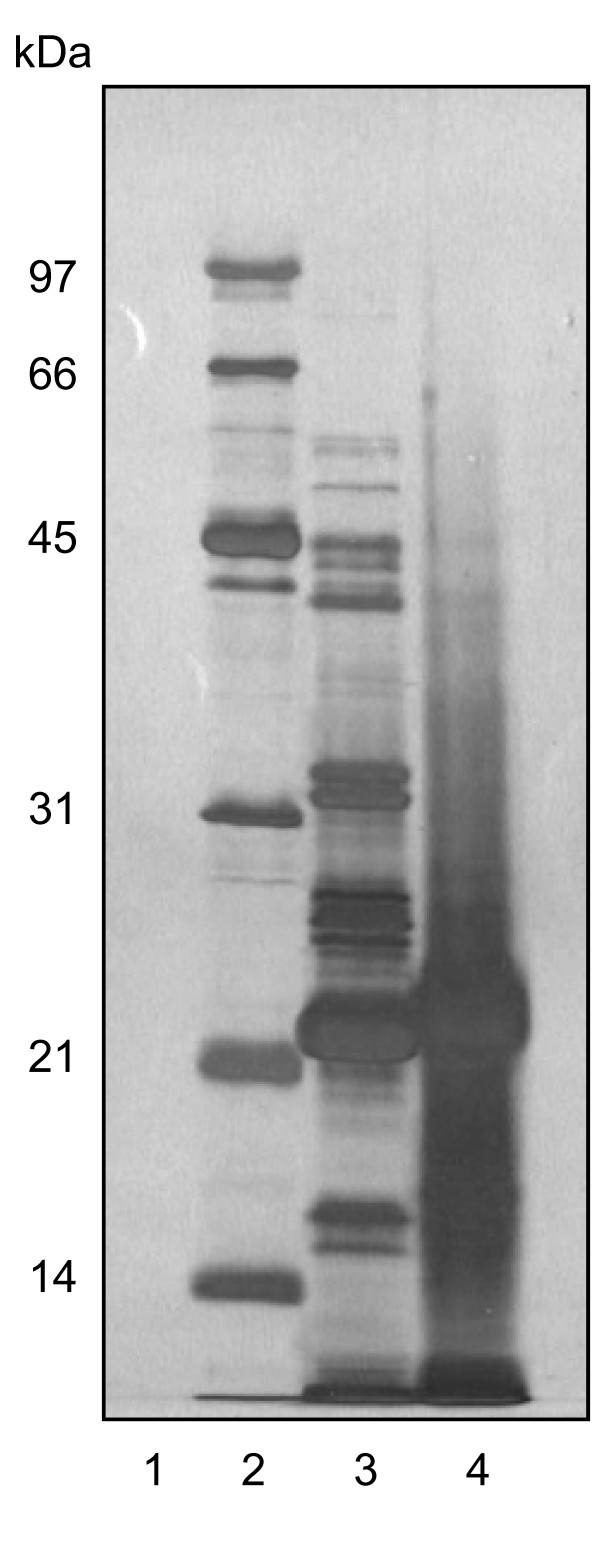
**Twelve percent SDS-PAGE analysis of HK PPD and SF-PPD**. (a) Gel stained with non-fixing silver stain. Lanes: 1, Sample buffer; 50 μL. 2. molecular weight standards (Silver Stain SDS-PAGE Standards Low Range, BIO-RAD). 3, SF-PPD; 12.5 μg. 4, HK-PPD; 12.5 ug.

The components of HK-PPD and SF-PPD were separated using SDS-PAGE and stained with either Coomassie Brilliant Blue (CBB) or silver stain so that the denaturing effect of autoclaving on *M. bovis *culture filtrate proteins could be visualized. Electrophoresis of HK-PPD resulted in the appearance of two blurred bands of approximately 10 and 23 kDa (Figure 1, lane 4). The profile of the HK-PPD was also dominated by the appearance of a large streak that commenced at approximately 45 kDa and increased in intensity at less than 25 kDa.

In comparison to HK-PPD, at least 35 bands could be visualized in SDS-PAGE of SF-PPD, many of which ranged from 23 to 80 kDa (Figure 1, lane 3). The two bands noted in PAGE of HK-PPD had corresponding bands of similar molecular weight in PAGE of SF-PPD however; the corresponding SF-PPD bands were discrete. Furthermore, the profile of SF-PPD lacked the streaked appearance noted in HK-PPD lanes.

### Western immunoblot comparison of HK-PPD and SF-PPD

**Figure 2:**
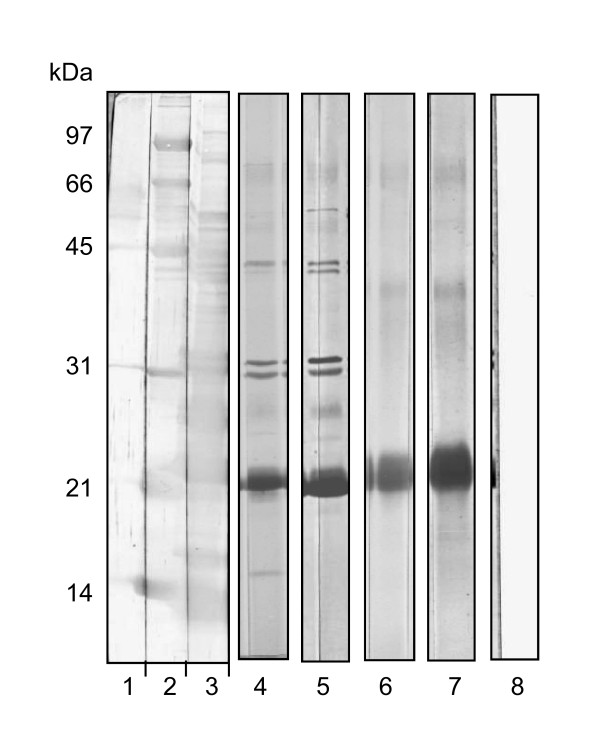
**Western blot analysis of HK-PPD and SF-PPD probed with either *M. bovis *infected or *M. bovis *sensitized cattle sera**. Twelve percent SDS-PAGE were performed followed by electrophoretic transfer of proteins to nitrocellulose. Lanes were cut into strips and individually stained or blotted. (a) Lanes 1, 2 and 3 were loaded with sample buffer, molecular weight standards (Silver Stain SDS-PAGE Standards Low Range, Bio-Rad) and 10 μg of SF-PPD respectively and were stained with Colloidal Gold (Bio-Rad) following transfer. Lanes 4 and 5 were loaded with 10 μg of SF-PPD and lanes 6 and 7 were loaded with 10 Φg of HK-PPD. Lanes 4 and 6 were blotted with *M. bovis *sensitized bovine sera (animal # 003); lanes 5 and 7 were blotted with *M. bovis *infected bovine sera (animal # 107). Lane 8 was loaded with 10 μg of HK-PPD and was blotted without sera (1° Ab) to act as a conjugate control.

The protein denaturing effect of autoclaving on *M. bovis *CFPs shown by PAGE and 2DE comparisons of HK-PPD *versus *SF-PPD tuberculin is suggested that the antigenicity of the SF-PPD differed from that of HK-PPD. Therefore, in order to investigate this further, SF-PPD and HK-PPD were compared by Western blot analysis using serum from *M. bovis *sensitized and infected cattle. Western blots of HK-PPD with either *M. bovis *infected or *M. bovis *sensitized sera revealed that the majority of antibody binding was restricted to one, predominant, poorly delineated 22 kDa band and to two additional faint bands of approximately 40 and 66 kDa (Figure 2, lane 6, 7). Contrary to SDS-PAGE analysis (Figure 1, lane 4), Western blots of HK-PPD did not detect antigen less than 20 kDa.

In comparison, Western blots of SF-PPD using the same *M. bovis *sensitized and *M. bovis *infected cattle sera, recognized more than 10 bands with molecular weights ranging from 16 - 90 kDa (Figure 2, lane 4, 5). As with blots of HK-PPD, few bands were detected at less than 20 kDa by either the *M. bovis *sensitized or infected sera, however, the sera did recognise several SF-PPD bands greater than 20 kDa which corresponded with bands observed in silver stained PAGE of SF-PPD (Figure 1). Small variations were observed in the Western blot banding patterns of SF-PPD among *M. bovis *infected and *M. bovis *sensitized sera. However, no consistent difference was observed in the Western blot banding patterns of SF-PPD probed with sera from the two groups of cattle, small variations were also observed among blots probed with sera obtained from individual cattle within both the M. bovis sensitized, and the *M. bovis *infected groups (data not shown).

### 2-DE analysis of SF-PPD and HK-PPD

**Figure 3:**
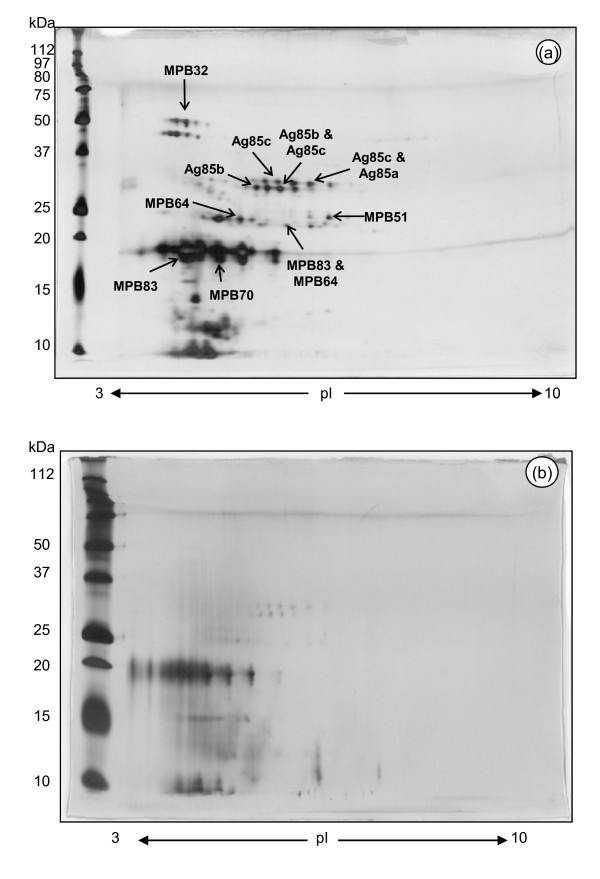
**2-DE analysis of SF-PPD and HK-PPD**. 10 μg of either SF-PPD (a) or HK-PPD (b) were loaded onto 17 cm pre-cast acrylamide strips with an immobilized pH range of 3-10 (ReadyStrip™; Bio-Rad). Following isoelectric focussing, the acrylamide strips were loaded into vertical 12% polyacrylamide gels with molecular weight standards (Precision Plus, Bio-Rad). The gels were stained with a non-fixing silver stain.

Although SDS-PAGE of SF-PPD resulted in improved delineation of proteins as compared to HK-PPD, it was hypothesized that two-dimensional SDS-PAGE (2-DE) would offer additional protein separation. Approximately 196 spots were enumerated from digitized 2DE images of SF-PPD by PDQuest 2D Analysis Software (Bio-Rad), the majority of which localized within a pI range of 4 - 6 and within a molecular weight range less than 80 kDa (Figure 3a).

In comparison to SF-PPD, 2D gels of HK-PPD were dominated by an indistinct smear which had a pI range of 3 - 5 and a molecular weight range of 0 - 80 kDa (Figure 3b). The majority of the HK-PPD components had molecular weights less than 25 kDa, and were blurred as compared to those of the SF-PPD gels. HK-PPD 2-DE gels also exposed approximately 30 spots from 5 - 15 kDa, in the 5.5 - 7.5 pI range that were not visualized in SF-PPD gels. A reduction from 50 μg to 10 μg of the HK-PPD protein level applied to 2-DE reduced the intensity of the smear and facilitated the detection of additional faint spots. However, only 69 individual spots were enumerated by PDQuest in 2-DE of HK-PPD (Figure 3b) as compared to the 196 individual spots enumerated in SF-PPD (Figure 3a).

### MASS SPEC analysis of nine SF-PPD spots

Nine 2DE SF-PPD spots were selected for MASS-SPEC analysis (Figure 3a). While the MASS-SPEC analysis potentially identified several mycobacterial proteins co-existing at each spot (TABLE 1), the SF-PPD spots were annotated based on the strength of association provided by the MASS-SPEC analysis (Mascot score) and by comparison to previously published 2-DE analysis of *Mycobacterium spp*. CFPs [10,12,15,16,25,26]. MPB32 was also annotated in Figure 3a based on its identical position and appearance to previously published 2DE analyses of mycobacterial CFPs [13,17,27].

**Table 1:** Results of MASS-SPEC analysis of 9 spots excised from 2DE gel of SF-PPD

SF-PPD spots for MASS SPEC analysis			Mascot Search Results		
	**~ 2DE spot location**		**Protein hits**		
	
**Designation**	**mass(kDa)**	**pl**	**Name**	**mass(kDa)**	**Score**

MPB83	22	4.2	**MPB83**	**22.2**	**471**
			
			CFP10	10.8	458
			
			MPB70	22.5	433
			
			MPB63	13.7	430
			
			ESAT-6	10.0	337

MPB70	22	4.7	**MPB70**	**22.5**	**588**
			
			MPB83	22.2	473
			
			Rv1314c	20.8	196
			
			CFP10	10.8	177
			
			MPB63	16.5	47

MPB64	25	4.5	**MPB64**	**25.1**	**733**
			
			MPB83	22.2	509
			
			MPB70	22.5	434
			
			*M.tb *protease	23.5	381
			
			lipoprotein LPPX	24.3	377

MPB83 & MPB64	25	5.4	Adenylate kinase	20.1	504
			
			**MPB83**	**22.2**	**466**
			
			**MPB64**	**25.1**	**366**
			
			Rv2557	24.7	356
			
			Ag85b	30.8	339

MPB51	27	6	**MPB51**	**31.1**	**699**
			
			enoyl-CoA hydratase	24.5	506
			
			MPB64	25.1	145
			
			MPB70	22.5	122
			
			Peptide of a 24 kDa immunoprotective protein	2.1	99

Ag85b	31	5	**Ag85b**	**30.8**	**609**
			
			*M.tb *protein MT3693	28.2	534
			
			Beta-1, glucanase precursor	32.2	329
			
			chaperonin GroEL	31.2	289
			
			29 kDa Ag	28.5	210

Ag85b & Ag85c	31	5.3	**Ag85b**	**30.8**	**610**
			
			Ag85c	31.2	417
			
			Ag85a	32.7	276
			
			Conserved membrane protein	27.3	107
			
			dehydrogenase/reductase	29.9	42

Ag85c	32	5.3	**Ag85c**	**33.1**	**632**
			
			esterase	34.0	611
			
			Ag85b	30.8	289
			
			Ag85a	32.8	191

Ag85c & Ag85a	32	5.7	**Ag85c**	**33.1**	**578**
			
			**Ag85a**	**32.8**	**351**
			
			Ag85b	30.8	309
			
			amidohydrolase	29.1	181
			
			TB15.3	15.3	133

Protein spots were excised from a CBB stained 2-DE gel of SF-PPD (200 μg; 50 μL of 4 μg/μL) and submitted to the Ottawa Institute of Systems Biology, University of Ottawa for MASS-SPEC analysis. In the table, the five most probable candidate proteins as determined by Mascot search results are listed for each submitted spot. Bold text is used to identify the most likely constituent or majority constituent protein for each submitted spot. Presumptive identifications are based on data provided by the Mascot search results and previously performed 2DE analysis of Mycobacterium spp. CFPs [15,20,27,28,32,35,40].

### 2-DE Western blot analysis of SF-PPD

**Figure 4:**
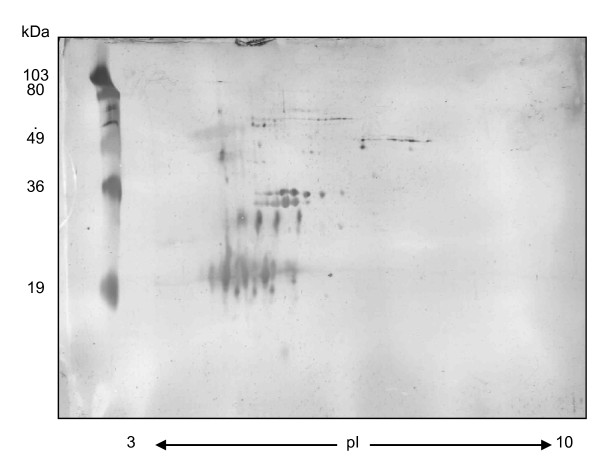
**2D-Western blot analysis of the antibody response to SF-PPD proteins in sera from an *M. bovis *sensitized bovine**. SF-PPD (200 μg) was loaded onto a 17 cm pre-cast acrylamide strip with an immobilized pH range of 3-10 (ReadyStrip™; Bio-Rad). Following isoelectric focussing, the acrylamide strip was loaded into vertical 12% polyacrylamide gels with molecular weight standards (Pre-stained - Low range, Bio-Rad). Protein was electrophoretically transferred to nitrocellulose and blotted with M. bovis sensitized cattle sera (animal # 893).

Following one-dimensional Western blot analyses on sera from both the *M. bovis *infected and *M. bovis *sensitized cattle groups, it became apparent that a two-dimensional (2D) Western blot approach would offer an improved visualization of the antibody response to SF-PPD antigens. The majority of the antibody response was observed with components located between 4 - 6 pI and within a molecular weight range of 20 - 36 kDa (Figure 4). In comparison to 2-DE of SF-PPD (Figure 3a), a series of spots at approximately 20 kDa and another cluster of spots at approximately 30 kDa appeared to be associated with the MPB70/MPB83 and Ag85 proteins detected by MASS-SPEC from 2-DE gels of SF-PPD (Figure 3a. A series of four spots at approximately 28 kDa with an approximate pI range of 4.5 - 5.5 was also associated with MPB64 and MPB83 by the same analysis. Antibody response was also detected within a 40 - 80 kDa and 4-8 pI range in the 2D Western blots. While the 40 - 80 kDa spots do not pair up with spots observed in 10 μg, 2-DE gels of SF-PPD (Figure 3a), they can be associated with spots observed on 2-DE gels loaded with 50 μg of SF-PPD (data not shown). In contrast to the 2-DE gels of SF-PPD, limited antibody response was observed to components at less than 20 kDa in 2D Western blots of SF-PPD.

Previous work in our laboratory showed that cattle artificially sensitized to *M. bovis *with an injection of heat killed *M. bovis *cells mounted a 30% greater average delayed type hypersensitivity (DTH) response to the intradermal application of *M. bovis *PPD tuberculin as compared to cattle experimentally infected with *M. bovis *(unpublished data). We hypothesized that immunological differences between these respective cattle models would be observed.

**Figure 5:**
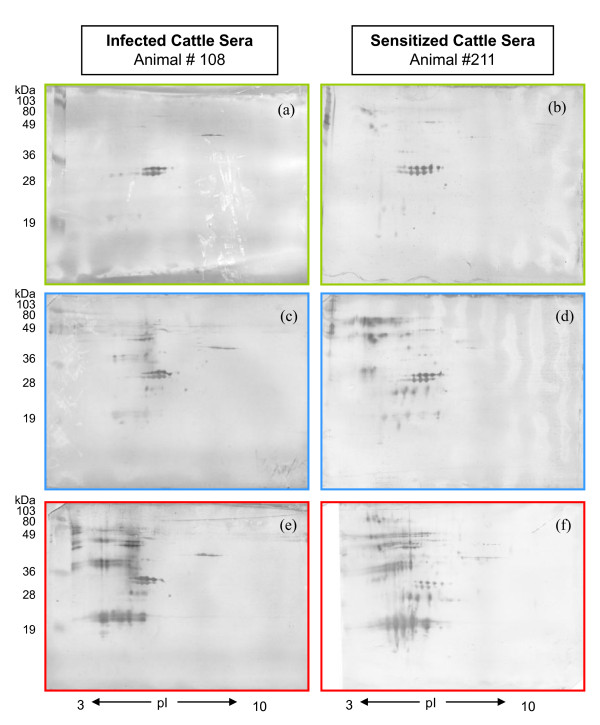
**2D-Western blot analysis of the antibody response to SF-PPD proteins in *M. bovis *infected and *M. bovis *sensitized cattle**. Sera were collected prior to sensitization (a) or infection (b); seven weeks post sensitization (c) or infection (d); and three weeks post tuberculin skin testing (e) and (f). Western blots (a), (c) and (e) are representative blots of the *M. bovis *sensitized cattle sera (N = 6). Western blots (b), (d) and (f) are representative blots of the *M. bovis *infected cattle sera (N = 6).

The serological response of six *M. bovis *infected and six *M. bovis *sensitized cattle were examined by 2D Western blot analysis (Figure 5). Sera from each animal were blotted at three time-points: pre-infection/pre-sensitization, seven weeks and thirteen weeks post sensitization or post infection (respectively) and three weeks post CITST. Every animal's pre-infection or pre-sensitization sera generated a background antibody response to SF-PPD antigens. While the background responses varied from animal to animal, the putative Ag85 complex was consistently observed with each animal's pre-infection or pre-sensitization sera (Figure 5a, b). A trend observed from the seven-week post infection/sensitization time-point indicated that the *M. bovis *sensitized cattle demonstrated a more intense antibody response and to additional *M. bovis *Ags, especially MPB70, MPB64 and MPB64/MPB83, as compared to the *M. bovis *infected cattle (Figure 5c, d, Additional files 1, 2, 3, 4, 5 and 6, FIG. S1 to S6). An overall, antibody-boosting effect was observed in the post-CITST time-point 2D Western blots of both the sensitized and infected cattle (Figure 5e, f). While the antibody response to SF-PPD protein spots at this time-point varied between individual animals within both the sensitized and infected cattle groups, each group appeared to respond similarly to the majority of *M. bovis *protein spots (Additional files 1, 2, 3, 4, 5 and 6, FIG. S1 to S6). No changes were observed from pre-injection to post-CITST in 2D Western blots of sera from a negative control animal which received a mineral oil/lanoline/saline injection (Additional file 7 FIG. S7).

## Discussion

Since the introduction of the tuberculin skin test (TST) by Koch in 1890 [2], tuberculin has been produced from heat-sterilized *M. bovis *culture filtrate and the TST remains the principal ante-mortem diagnostic test for bovine tuberculosis worldwide. Remarkably, the composition of tuberculin and the antigenicity of its components remain largely unknown even though it is now recognized that 2-4 week old, non-heated *M. bovis *culture filtrate is composed of more than 800 CFPs [12]. Previous attempts to separate the active components of tuberculin by a variety of fractionation [3-8] and electrophoretic techniques [9,28] have led to equivocal results and difficulty in interpretation, most likely due to the observation that heat killing during tuberculin production leads to profound structural and possibly other un-characterized changes to the mycobacterial CFPs.

In this study, the effect of autoclaving on mycobacterial CFPs is readily evidenced by our observation that there is at least a three-fold increase in additional spots that can be enumerated in 2-DE gels of non-heated SF-PPD as compared to HK-PPD. Furthermore, the differences observed between gels of SF-PPD and HK-PPD do not appear to be restricted to protein denaturation as both SF-PPD and HK-PPD proteins are also denatured by the SDS-PAGE protocol which uses a combination of heat, SDS and 2-ME treatment prior to electrophoresis. Therefore, the effects of heat and pressure due to the autoclave process are likely responsible for the creation of tuberculo-protein peptide fragments in the HK-PPD preparation which appear as streaks or smears on PAGE or 2DE gels. These results are consistent with previous attempts to characterize the constituents of tuberculin [2,9] all of which professed difficulty delineating protein bands from autoclaved preparations of tuberculin.

Although the majority of HK-PPD proteins appeared blurred as compared to of SF-PPD in 2-DE gels, the delineation of more than 60 individual protein spots in 2-DE of HK-PPD (Figure 3b) suggested that PPD tuberculin may contain a greater number of whole tuberuclo-proteins than previously believed [6,9,29,30]. The protein group that appeared to have the greatest resilience to the effects of autoclaving was the MPB70 and MPB83 protein group. This result is in accordance with previous studies involving autoclaved *M. bovis *culture filtrate [9], where the heat stability of the distinct but highly homologous MPB70 and MPB83 proteins was attributed to the presence of identical 133 amino acid disulfide loops and the formation of stable aggregates in the culture fluid [31,32]. The detection of MPB32 in HK-PPD (faintly visible in Additional file 8 FIG. S8) is especially interesting as this protein was described by Nagai *et al*. as the most heat labile protein as compared to MPB64 and the Ag85 complex proteins [10].

The suspected presence of heat liable *M. bovis *CFPs in PPD tuberculin therefore stresses the importance of conformity between bulk lots of PPD tuberculin with respect to the heat kill parameters. Reduced autoclave time and/or pressure would likely increase the concentration of whole proteins whereas increased time and pressure may reduce even the peptide fragments to non-antigenic subunits. This has been shown by Borsuk et al [33] who reported that *M. bovis *protein rV3874 was the most abundant tuberculo-protein preset in *M. bovis *PPD tuberculin produced in the UK while the same protein did not rank is the top ten most abundant tuberculo-proteins found in *M. bovis *PPD produced in Brazil. Although both the UK and Brazilian PPDs were derived from *M. bovis *AN5, the heat kill parameters used to produce the two tuberculins differed greatly While Trevedi et al. [34] reported no significant difference between the DTH response elicited by three-week-old *M. bovis *CFPs as compared to those provoked by *M. bovis *PPD tuberculin in *M. bovis *infected cattle, the immunological significance of the concentration of *M. bovis *peptide fragments in PPD tuberculin remains to be elucidated.

The 2-DE gels of HK-PPD also displayed protein spots which were not observed in 2-DE of SF-PPD (Figure 3b). These HK-PPD spots were located between 10 - 15 kDa and possessed approximate pIs of 6 - 8. While these spots could represent additional cytoplasmic proteins released into HK-PPD through the autoclave process, we did not detect antibody recognition to these protein spots in corresponding locations using Western blot analysis (data not shown). Due to the low molecular weight of these proteins and the failure to detect these spots in 2-DE of SF-PPD, it is suspected that these spots actually represented complexes of CFPs which were reduced to peptide fragments by the autoclaving process. The immunological significance of these protein spots with respect to the tuberculin skin test response has yet to be determined.

The majority of approximately 200 distinct protein spots observed on 2-DE gels of SF-PPD were easily visualized and clearly delineated as compared to the smeared presentation of HK-PPD. Although this number falls well short of the 800 plus protein spots reported to exist in *M. bovis *culture filtrate [12], the MASS-SPEC results from this study indicated that each protein spot actually contained a mixture of multiple *M. bovis *CFPs (TABLE 1). Another potential reason for this difference in detected protein spots is that our preparation of *M. bovis *CFPs was obtained from 9-week-old culture supernatant in order to characterize the actual constituents that make up PPD tuberculin whereas other studies used shorter culture times for their CFP preparations [12,35]. The concentration of CFPs changes as the culture time increases, with the more stable proteins, for example MPB70/83, building up over time while others, such as MPB64 tend to degrade in the culture supernatant [14].

One-dimensional Western blots of sera from *M. bovis *infected or sensitized cattle onto HK-PPD revealed that the majority of antibody binding to HK-PPD was largely restricted to a 22-kDa band. However, when the same sera were blotted to SF-PPD additional bands were visualized. The 22 kDa band observed in blots of HK-PPD has previously been interpreted to indicate reactivity to both MPB70 and MPB83, however, the two additional weak bands with apparent weights of 40 and 66 kDa, (Figure 2 lanes 4 & 5) may also have consisted of MPB70 and MPB83 as these proteins have previously been shown to present as dimers and trimers in Western blots of *M. bovis *CFPs [31,32]. The streaked appearance of HK-PPD Western blots is suspected to result from the HK-PPD peptide fragments which are recognized by antibodies in the *M. bovis *sensitized/infected sera. The background effect resulting from these peptide fragments likely masked the antibody responses to small quantities of whole HK-PPD proteins. Therefore, although the antibodies in *M. bovis *sensitized sera may have recognised peptide fragments originating from a multitude of HK-PPD antigens, we were unable to ascertain from which *M. bovis *CFPs the peptide fragments originated.

The occurrence of antibody recognition of SF-PPD antigen in all twelve pre-infection/sensitization cattle sera was unexpected (Additional files 1, 2, 3, 4, 5 and 6, FIG. S1-S6), especially since all twelve animals tested negative on the pre-screening INF-(based *in vitro *test for bovine tuberculosis (results not shown). Although the pre-existing antibody level to SF-PPD antigens varied between animals, all twelve cattle recognized a similar group of SF-PPD proteins in 2D Western blot analysis which corresponded to the Ag85 complex proteins identified by MASS-SPEC analysis (Figure 3a, Figure 5a, b). Interestingly, this background antibody level did not appear to be correlated to animal age or to isolation of housing facility. Cattle designated for the infection trial were purchased from a single farm at four months of age and maintained within a Bio-containment Level III facility while cattle designated for the *M. bovis *sensitization trial were purchased from various local farms and maintained to approximately 1.5 years of age in an open air, large animal facility prior to the initiation of the study.

Ag85 complex proteins are not specific to *Mycobacterium tuberculosis *complex strains but are also known to be expressed by most environmental mycobacteria [36,37]. *M. avium *and *M. bovis *Ag85 complex proteins reportedly share 99% homology at the protein levels [38] and both Santema et al and Borsuk et al have identified Ag85 complex proteins in *M. avium *PPD by MASS-SPEC [33,39]. Therefore it is not surprising that cross reactive epitopes have been shown between the respective Ag85 complexes [10]. Both Mustafa *et al*. and Amadori *et al. *[30,40] credited a prior exposure to environmental mycobacteria as the basis for low levels of Ag85 complex antibody recognition in *M. bovis *negative cattle. Espitia *et al*. and Al-Attiyah *et al. *[41,42] also attributed environmental mycobacteria as a potential determinant for Ag85 complex antibody recognition in the sera of tuberculosis-free humans. Therefore, the presence of Ag85 in PPD tuberculin may contribute to the relatively low specificity of the TST. An analysis of *M. avium *PPD tuberculin by 2DE and 2D Western blot analysis may help elucidate other potentially cross-reactive proteins.

Additional SF-PPD antigenic determinants were detected from 2D Western blots of sera from *M. bovis *sensitized cattle as compared to *M. bovis *infected cattle at seven weeks post *M. bovis *infection/sensitization. Little difference was noted in the antibody response to SF-PPD antigens in sera taken at seven weeks post infection as compared to the pre-infection sera. This was in accordance with other previous *M. bovis *infection studies in which antibody response to *M. bovis *CFP antigens were initially detected between 7-10 weeks post infection [43,44]. One reason for the noted difference between the infected and sensitized cattle in our study may be explained by the antigenic dose. Cattle sensitized with a 20 mg intramuscular injection of heat killed *M. bovis *cells may have been exposed to a higher dose of *M. bovis *CFPs in the first seven weeks of this study as compared to the infected cattle that received 1500 CFU of live M. bovis by intra-tracheal inoculation.

Application of the CITST resulted in a dramatic boosting of the antibody response to SF-PPD in both the *M. bovis *infected and *M. bovis *sensitized cattle. While several other studies have indicated a similar boosting effect caused by tuberculin for both the humoral [1,9,20,44,45] and cellular [1,19,46] immune responses of *M. bovis *infected cattle, the precise mechanisms by which this occurs remains to be determined [35]. Harboe *et al. *[43] originally hypothesized that the marked increase in antibody response following skin testing in cattle was principally due to the presence of native MPB70 in *M. bovis *PPD tuberculin. Harboe *et al*. (1990) further postulated that a similar antibody boosting effect following a TST was typically not observed in human tuberculosis patients due to the minimal amount of MPB70 present in *M. tuberculosis *PPD tuberculin [43]. This is contradictory to our results that indicated a general increase in antibody response to several SF-PPD proteins. The disparity of results between Harboe *et al. *[43] and the findings in this study may be explained, in part, by the different methods used to analyse the sera. Our use of 2D Western blots presumably provided an increased separation and sensitivity of the *M. bovis *CFPs as compared to the one dimensional Westerns performed by Harboe et al. [43]. The increased separation of *M. bovis *CFPs may have therefore permitted a more precise analysis of the antigen recognition by serum antibodies. Our observations do concur with the results of an *M. bovis *infection study by Lyashchenko *et al*. which indicated that the application of a TST induced an antibody boost to several other *M. bovis *CFPs proteins including MPB64 [1]. While the mechanism of this boosting effect remains to be completely characterized, it is likely that peripheral B-cells are stimulated to produce antibodies to predominantly linear epitopes of the soluble PPD tuberculin proteins following cognate interactions with T-cells.

We did not observe significant antibody responses to the SF-PPD proteins of molecular weights less than 20 kDa in *M. bovis *infected or sensitized cattle sera. This result was in contradiction to findings by other researchers who showed that both ESAT-6 and CFP10 (which are reported to resolve at approximately 8 and 12 kDa respectively in 2-DE analyses of mycobacterial CFPs [17], have previously been shown to elicit an antibody boosting effect in *M. bovis *infected cattle following skin testing [1,9,20,44]. Since ESAT-6 is known to be secreted in the early stages of *M. bovis *infection [47-50], it is suspected that the *M. bovis *infected cattle would have elicited an antibody response to ESAT-6 and/or CFP10. However, our Western blot analysis was not able to detect significant antibody response at 8 and/or 12 kDa. Of note, ESAT-6 and CFP-10 proteins were detected in 2-DE of SF-PPD by MASS-SPEC analysis at 22 kDa, 4.2 & 4.7 pI respectively (Additional files 1, 2, 3, 4, 5 and 6, FIG. S1-S6). While this indicates that ESAT-6 and CFP-10 are present in SF-PPD, it does not allude to their respective concentrations. In addition the 9 week long propagation of *M. bovis *cultures likely does not favour maximum yield of ESAT-6 and CFP-10 as the proteins with increased structural integrity, for example MPB70/83, are more suited to persist in the culture fluid following secretion. Furthermore, the dialysis and concentration of *M. bovis *culture filtrate at 10 kDa may have resulted in the partial loss of these two proteins from both HK-PPD and SF-PPD preparations. Therefore, the detection of ESAT-6 and CFP-10 in SF-PPD at 22 kDa is likely an example of the sensitivity of MASS-SPEC as opposed to the key location for these proteins.

The use of a more specific/sensitive technique such as multi-antigen print immunoassay (MAPIA) which employs recombinant mycobacterial proteins, may be required to detect serum antibodies to ESAT-6 and CFP-10 as has been used previously [1,19,20,46]. While our Western blotting technique involved denaturation of protein by SDS and 2ME, thereby restricting our analysis to linear epitopes, MAPIA and select (-INF based diagnostic tests use recombinant proteins in a native conformation. Therefore, serum antibodies specific for conformational epitopes may recognise the native conformation of the antigens used in MAPIA that could thereby alter the antibody recognition for a given tuberculo-protein. While the presence and quantity of both ESAT-6 and CFP10 in SF-PPD may be determined by future studies using monoclonal antibodies specific for the respective proteins or another more sensitive method, the existence of an antibody response to either ESAT-6 or CFP10 from *M. bovis *infected/sensitized cattle cannot be concluded from this study.

## Conclusions

*M. bovis *PPD tuberculin has been used for more than a century to control one of the most devastating bacterial diseases of all times however the actual antigenic constituents of *M. bovis *PPD tuberculin and the immunological events initiated by its use have yet to be fully understood. The main purpose of this study was to characterize the antigenic constituents of *M. bovis *CFPs and to compare the antibody response of *M. bovis *infected cattle to that of cattle artificially sensitized to *M. bovis *by 2D Western blot analysis. Our 2-DE analysis of HK-PPD revealed that PPD tuberculin likely consists of a multitude of whole *M. bovis *proteins in addition to peptide fragments originating from *M. bovis *CFPs.

We concluded that it is the heat sterilization of the *M. bovis *CFPs which caused the severe structural changes and protein fragmentation observed in the HK-PPD *M. bovis *proteins. This work also suggested that *M. bovis *infected cattle and cattle artificially sensitized to *M. bovis *with an injection of heat-killed cells exhibited similar antibody response to the *M. bovis *antigens under study. While this study compares the humoral immune response of *M. bovis *infected and *M. bovis *sensitized cattle, future comparisons of their cellular immune responses may lead an *M. bovis *sensitization method which would provide a consistent immune response similar to that of an experimentally *M. bovis *infected animals. The development of an accurate, non-infectious bovine tuberculosis model would reduce the complexity and bio-containment risks associated with live *M. bovis *studies.

## Authors' contributions

BR performed all of the laboratory work as a partial fulfillment of his MSc degree at the University of Ottawa. BR wrote the manuscript and the two senior authors (LF and NS) corrected and approved the manuscript. All authors participated in the conception of the study and participated in its design and coordination. All authors read and approved the final manuscript.

## Supplementary Material

Additional file 1 **Fig. S1**. Western blot analysis of the antibody response to SF-PPD proteins in cattle prior to *M. bovis *sensitization. Click here for file

Additional file 2 **Fig. S2**. Western blot analysis of the antibody response to SF-PPD proteins in cattle at seven weeks post *M. bovis *sensitization. Click here for file

Additional file 3 **Fig. S3**. Western blot analysis of the antibody response to SF-PPD proteins in *M. bovis *sensitized cattle post CITST. Click here for file

Additional file 4 **Fig. S4**. Western blot analysis of the antibody response to SF-PPD proteins in cattle prior to *M. bovis *infection. Click here for file

Additional file 5 **Fig. S5**. Western blot analysis of the antibody response to SF-PPD proteins in cattle at seven weeks post *M. bovis *infection. Click here for file

Additional file 6 **Fig. S6**. Western blot analysis of the antibody response to SF-PPD proteins in *M. bovis *infected cattle post CITST. Click here for file

Additional file 7 **Fig. S7**. Western blot analysis of the antibody response to SF-PPD proteins in negative control cattle #9246. (not sensitized to, or infected with *M. bovis*.). (a) Pre-injection of mineral oil/lanoline; (b) seven weeks post mineral oil/lanoline injection; (c) Post CITST. Click here for file

Additional file 8 **Fig. S8**. Silver stained 2-DE analysis of HK-PPD depicting MPB32. 50 μg of HK-PPD was loaded onto 17 cm pre-cast acrylamide strip with an immobilized pH range of 3-10 (ReadyStrip™; Bio-Rad). Following isoelectric focussing, the acrylamide strip was loaded into a vertical 12% polyacrylamide gel with molecular weight standard (Silver Stain SDS-PAGE Standards, Low Range, Bio-Rad). Click here for file
