# Supplementary figures and images for: Antibody response to a sterile filtered PPD tuberculin in M. bovis infected and M. bovis sensitized cattle
Source: BMC Vet Res. 2010 Nov 9;6:50. doi: 10.1186/1746-6148-6-50 (PMC2994848; doi:10.1186/1746-6148-6-50)

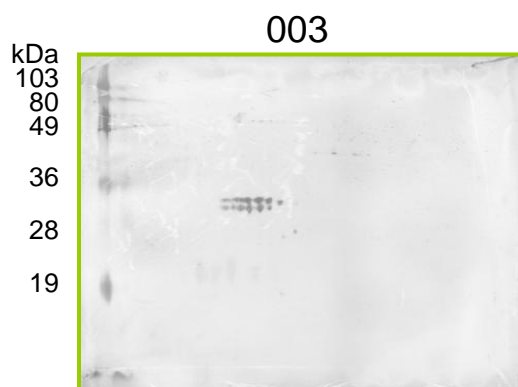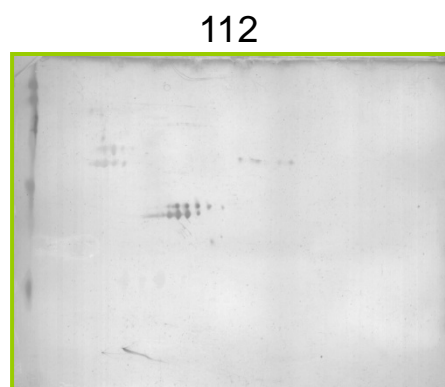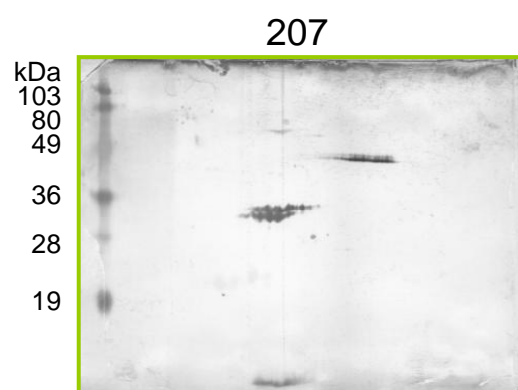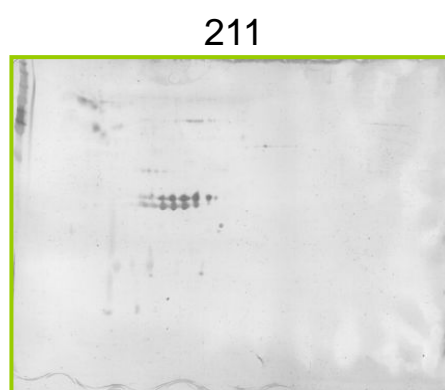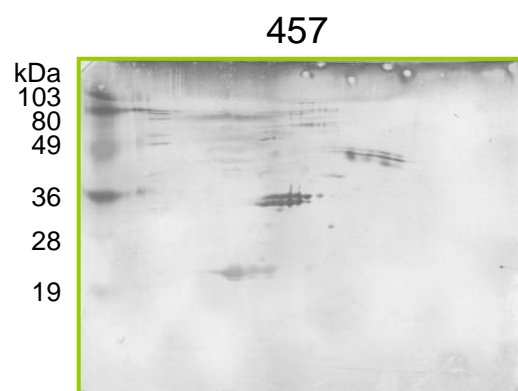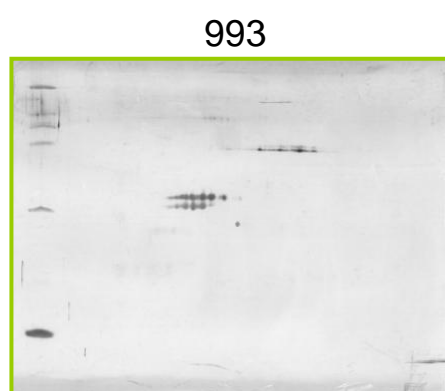

3 ← pl → 10

3 ← pl → 10

Supplement: Additional file 1 — Fig. S1. Western blot analysis of the antibody response to SF-PPD proteins in cattle prior to M. bovis sensitization. [file 1746-6148-6-50-S1.PDF]

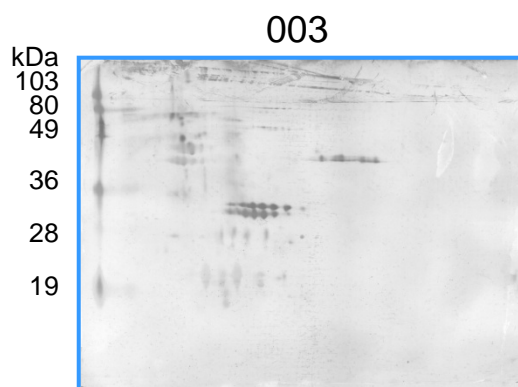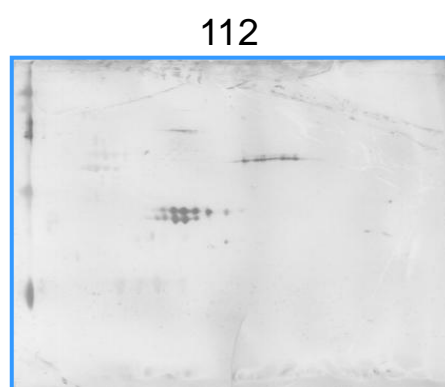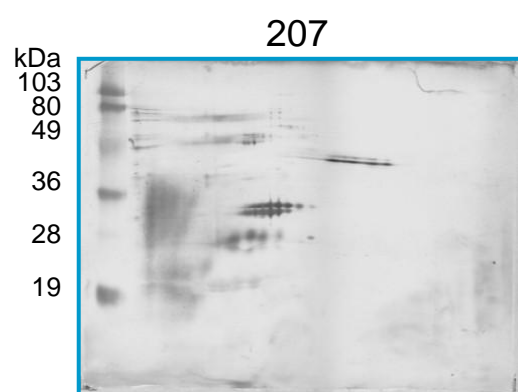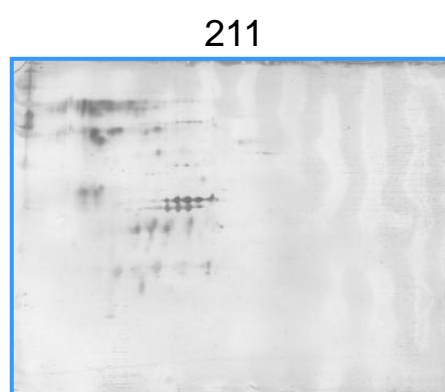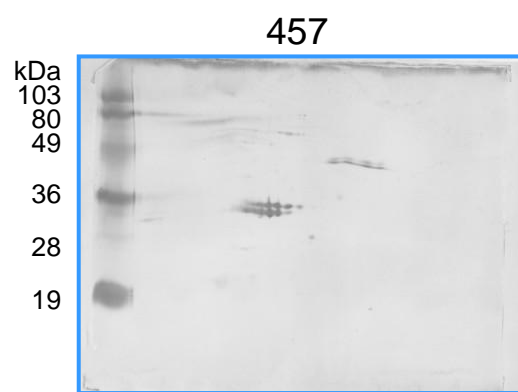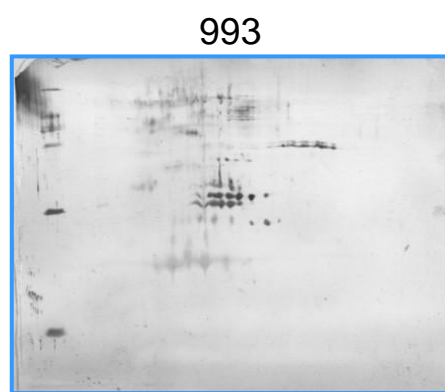

3 ← pI → 10

3 ← pI → 10

Supplement: Additional file 2 — Fig. S2. Western blot analysis of the antibody response to SF-PPD proteins in cattle at seven weeks post M. bovis sensitization. [file 1746-6148-6-50-S2.PDF]

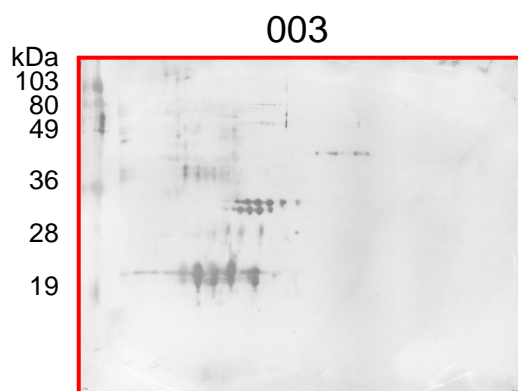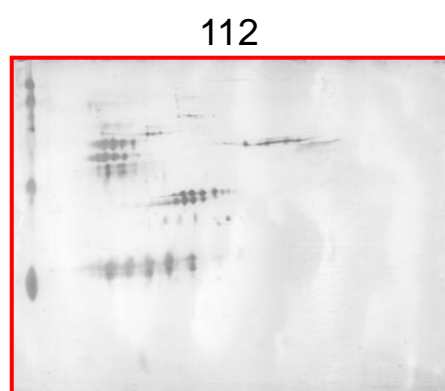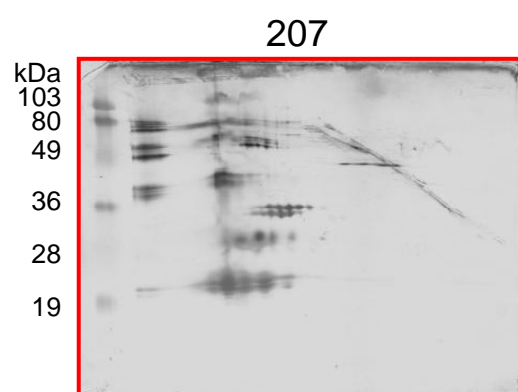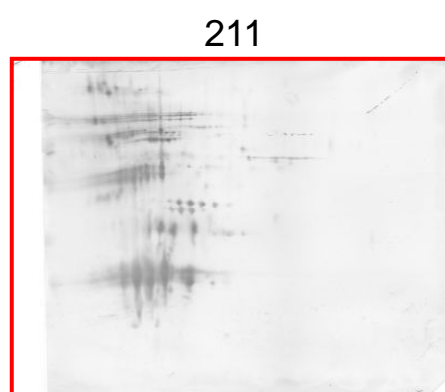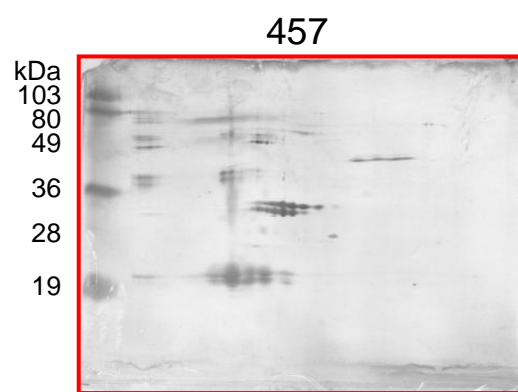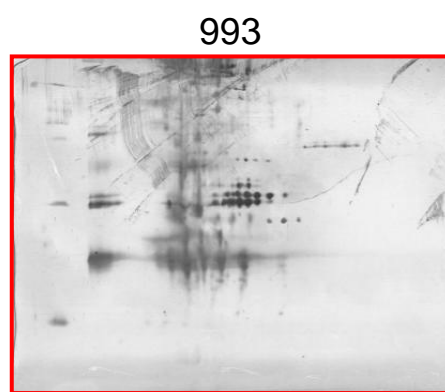

3 ← pl → 10

3 ← pl → 10

Supplement: Additional file 3 — Fig. S3. Western blot analysis of the antibody response to SF-PPD proteins in M. bovis sensitized cattle post CITST. [file 1746-6148-6-50-S3.PDF]

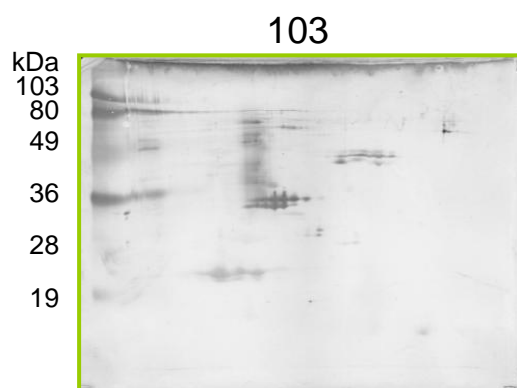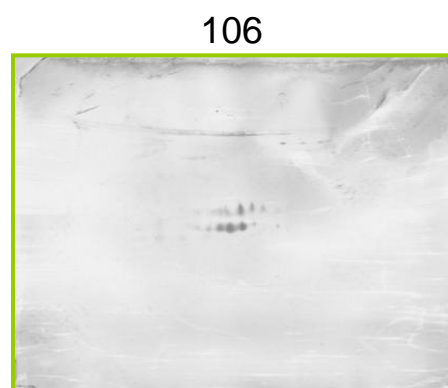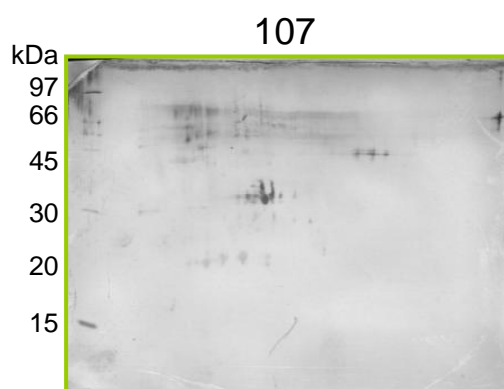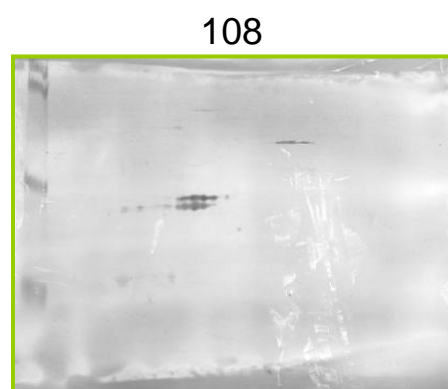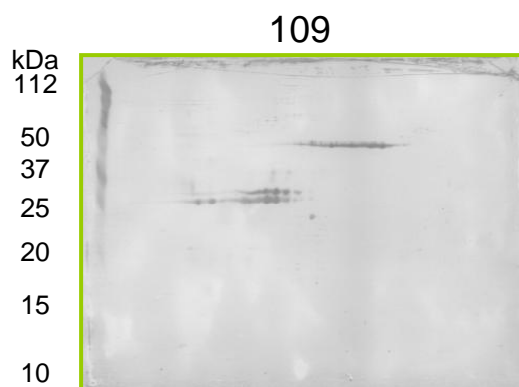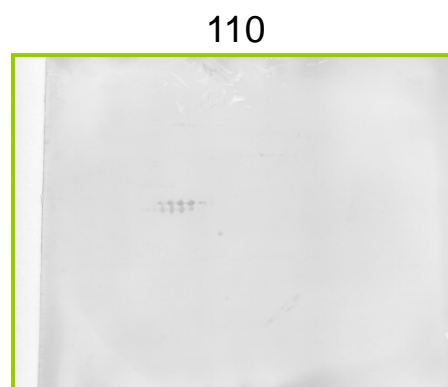

3 ← pI → 10

3 ← pI → 10

Supplement: Additional file 4 — Fig. S4. Western blot analysis of the antibody response to SF-PPD proteins in cattle prior to M. bovis infection. [file 1746-6148-6-50-S4.PDF]

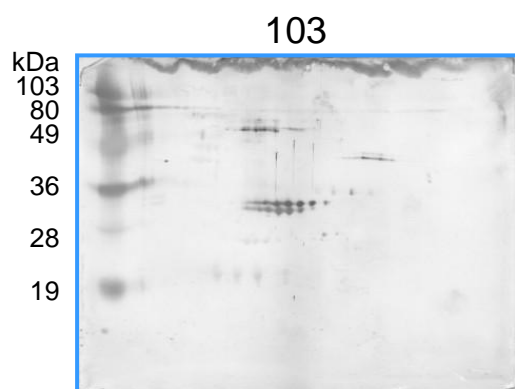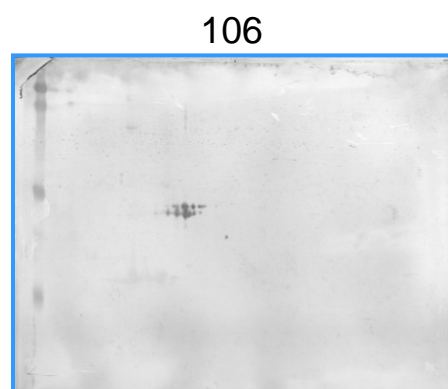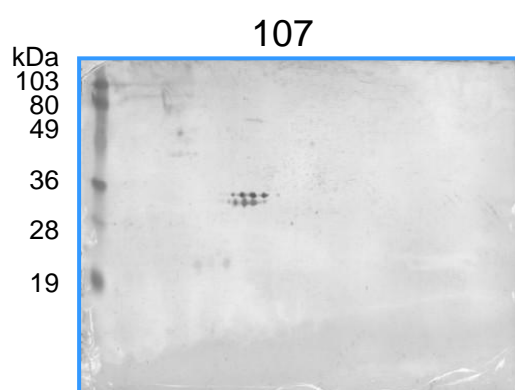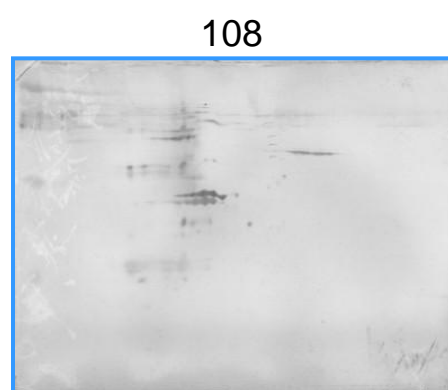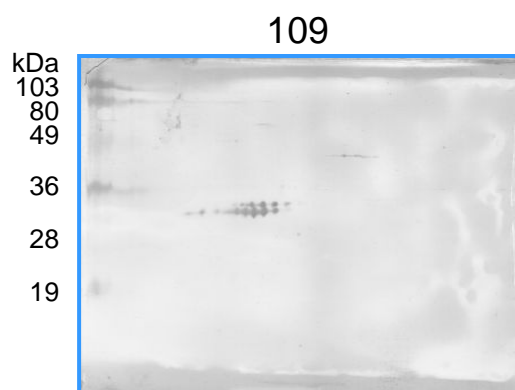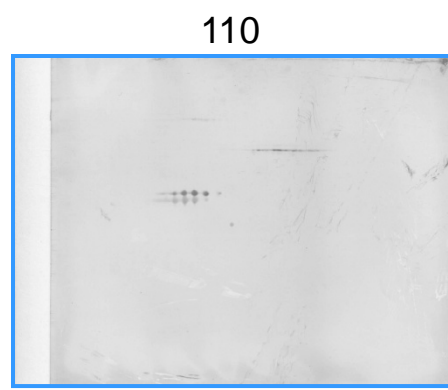

3 ← pl → 10

3 ← pl → 10

Supplement: Additional file 5 — Fig. S5. Western blot analysis of the antibody response to SF-PPD proteins in cattle at seven weeks post M. bovis infection. [file 1746-6148-6-50-S5.PDF]

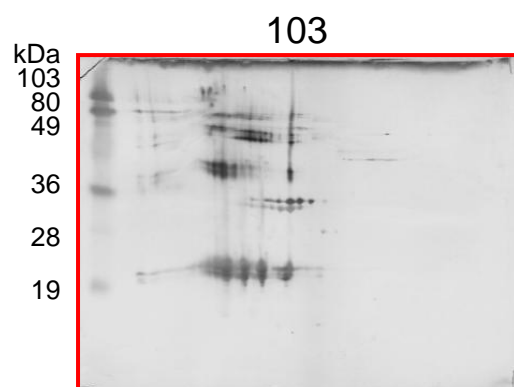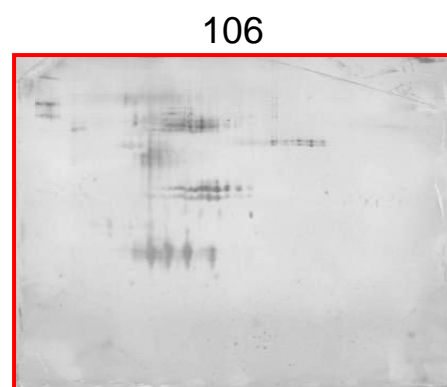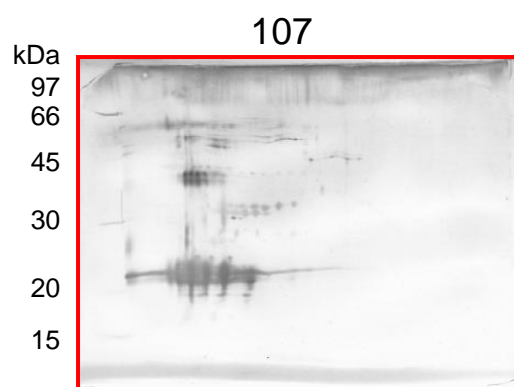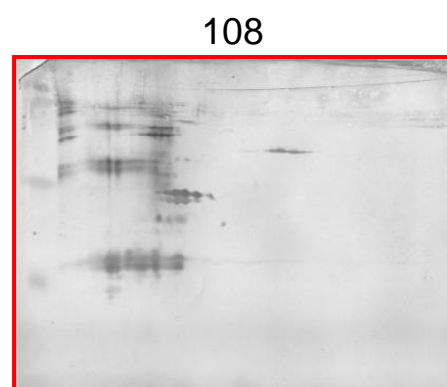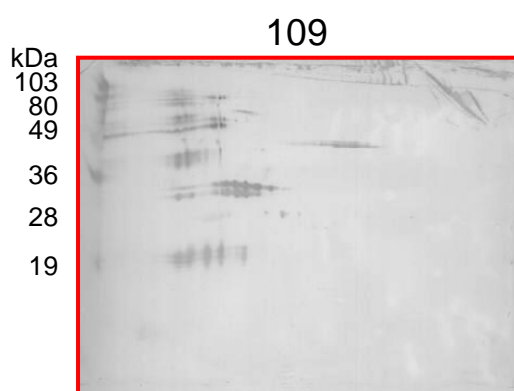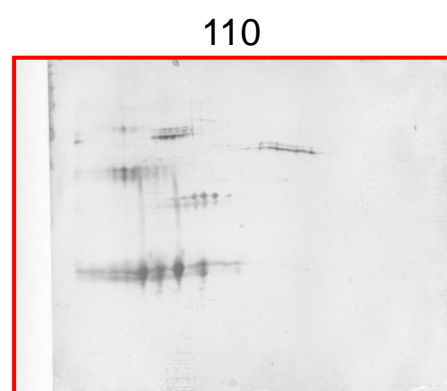

3 ← pI → 10

3 ← pI → 10

Supplement: Additional file 6 — Fig. S6. Western blot analysis of the antibody response to SF-PPD proteins in M. bovis infected cattle post CITST. [file 1746-6148-6-50-S6.PDF]

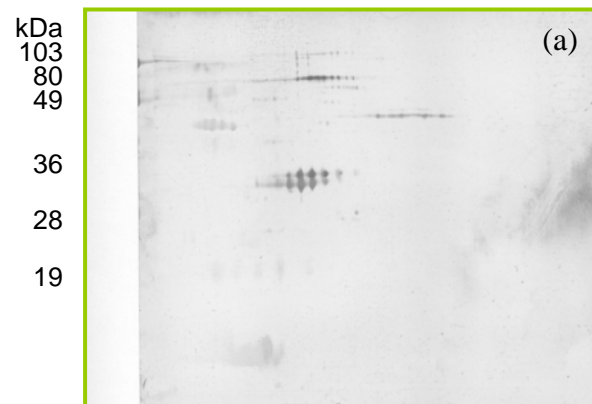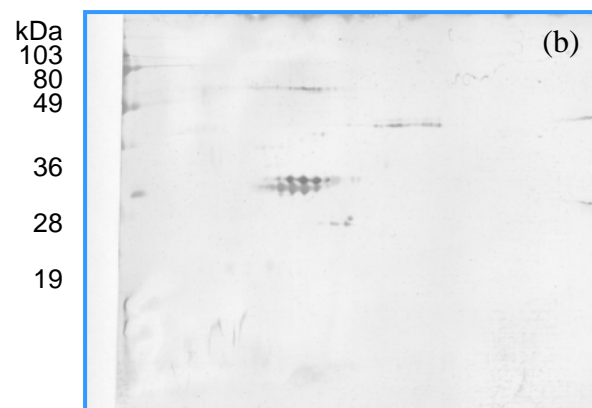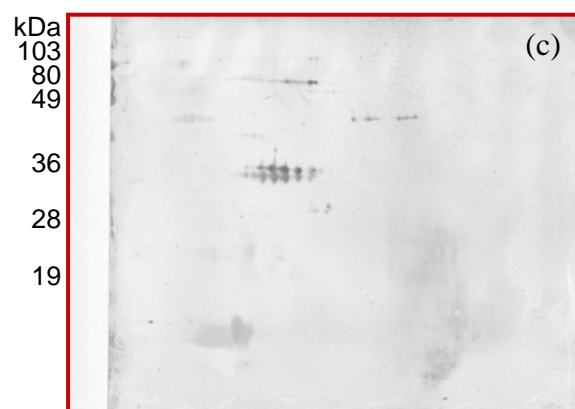

3 ← pI → 10

Supplement: Additional file 7 — Fig. S7. Western blot analysis of the antibody response to SF-PPD proteins in negative control cattle #9246. (not sensitized to, or infected with M. bovis.). (a) Pre-injection of mineral oil/lanoline; (b) seven weeks post mineral oil/lanoline injection; (c) Post CITST. [file 1746-6148-6-50-S7.PDF]

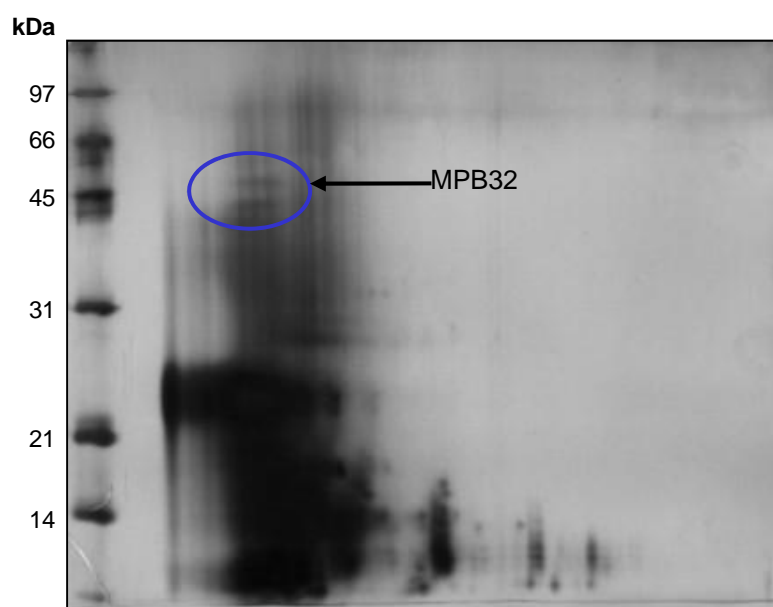

Supplement: Additional file 8 — Fig. S8. Silver stained 2-DE analysis of HK-PPD depicting MPB32. 50 μg of HK-PPD was loaded onto 17 cm pre-cast acrylamide strip with an immobilized pH range of 3-10 (ReadyStrip™; Bio-Rad). Following isoelectric focussing, the acrylamide strip was loaded into a vertical 12% polyacrylamide gel with molecular weight standard (Silver Stain SDS-PAGE Standards, Low Range, Bio-Rad). [file 1746-6148-6-50-S8.PDF]
